# RUNX3 Has an Oncogenic Role in Head and Neck Cancer

**DOI:** 10.1371/journal.pone.0005892

**Published:** 2009-06-12

**Authors:** Takaaki Tsunematsu, Yasusei Kudo, Shinji Iizuka, Ikuko Ogawa, Tsuyoshi Fujita, Hidemi Kurihara, Yoshimitsu Abiko, Takashi Takata

**Affiliations:** 1 Division of Frontier Medical Science, Department of Oral and Maxillofacial Pathobiology, Graduate School of Biomedical Sciences, Hiroshima University, Hiroshima, Japan; 2 Center of Oral Clinical Examination, Hiroshima University Hospital, Hiroshima, Japan; 3 Division of Frontier Medical Science, Department of Periodontal Medicine, Graduate School of Biomedical Sciences, Hiroshima University, Hiroshima, Japan; 4 Department of Biochemistry, School of Dentistry at Matsudo, Nihon University, Tokyo, Japan; Karolinska Institutet, Sweden

## Abstract

**Background:**

Runt-related transcription factor 3 (RUNX3) is a tumor suppressor of cancer and appears to be an important component of the transforming growth factor-beta (TGF-ß)-induced tumor suppression pathway. Surprisingly, we found that RUNX3 expression level in head and neck squamous cell carcinoma (HNSCC) tissues, which is one of the most common types of human cancer, was higher than that in normal tissues by a previously published microarray dataset in our preliminary study. Therefore, here we examined the oncogenic role of RUNX3 in HNSCC.

**Principal Findings:**

Frequent RUNX3 expression and its correlation with malignant behavior were observed in HNSCC. Ectopic RUNX3 overexpression promoted cell growth and inhibited serum starvation-induced apoptosis and chemotherapeutic drug induced apoptosis in HNSCC cells. These findings were confirmed by RUNX3 knockdown. Moreover, RUNX3 overexpression enhanced tumorsphere formation. RUNX3 expression level was well correlated with the methylation status in HNSCC cells. Moreover, RUNX3 expression was low due to the methylation of its promoter in normal oral epithelial cells.

**Conclusions/Significance:**

Our findings suggest that i) RUNX3 has an oncogenic role in HNSCC, ii) RUNX3 expression observed in HNSCC may be caused in part by demethylation during cancer development, and iii) RUNX3 expression can be a useful marker for predicting malignant behavior and the effect of chemotherapeutic drugs in HNSCC.

## Introduction

RUNX3/AML2/PEBP2C/CBFA3 is a transcription factor and one of the Runt-related (RUNX) family. Three members of the Runx family genes, *RUNX1*, *RUNX2* and *RUNX3*, and related gene, *CBFB/Pebpb2*, are all known as the developmental regulators and have been shown to be important in human cancers [1]. RUNX3 was originally cloned as AML2 and is localized on chromosome 1p36.1 [2]. RUNX3 has multiple functions and was at first reported to correlate with the genesis and progression of human gastric cancer as a tumor suppressor [2]. Runx3-null mice exhibit hyperplasia of gastric mucosa as a result of stimulated proliferation and suppressed apoptosis of epithelial cells [3]. In fact, RUNX3 is inactivated in more than 80% of human gastric cancer by gene silencing and protein mislocalization [4]. Besides gastric cancer, it has been reported that reduced expression of RUNX3 was observed in various cancers including bladder, liver, colorectal and lung cancers [5]–[8]. In these tumors, reduced expression of RUNX3 was frequently caused by CpG island hypermethylation [9]. Moreover, point mutations of *RUNX3* were observed in certain type of human cancers including gastric and bladder cancers [3], [5]. Taken together, RUNX3 acts as a tumor suppressor in various cancers.

**Figure 1:**
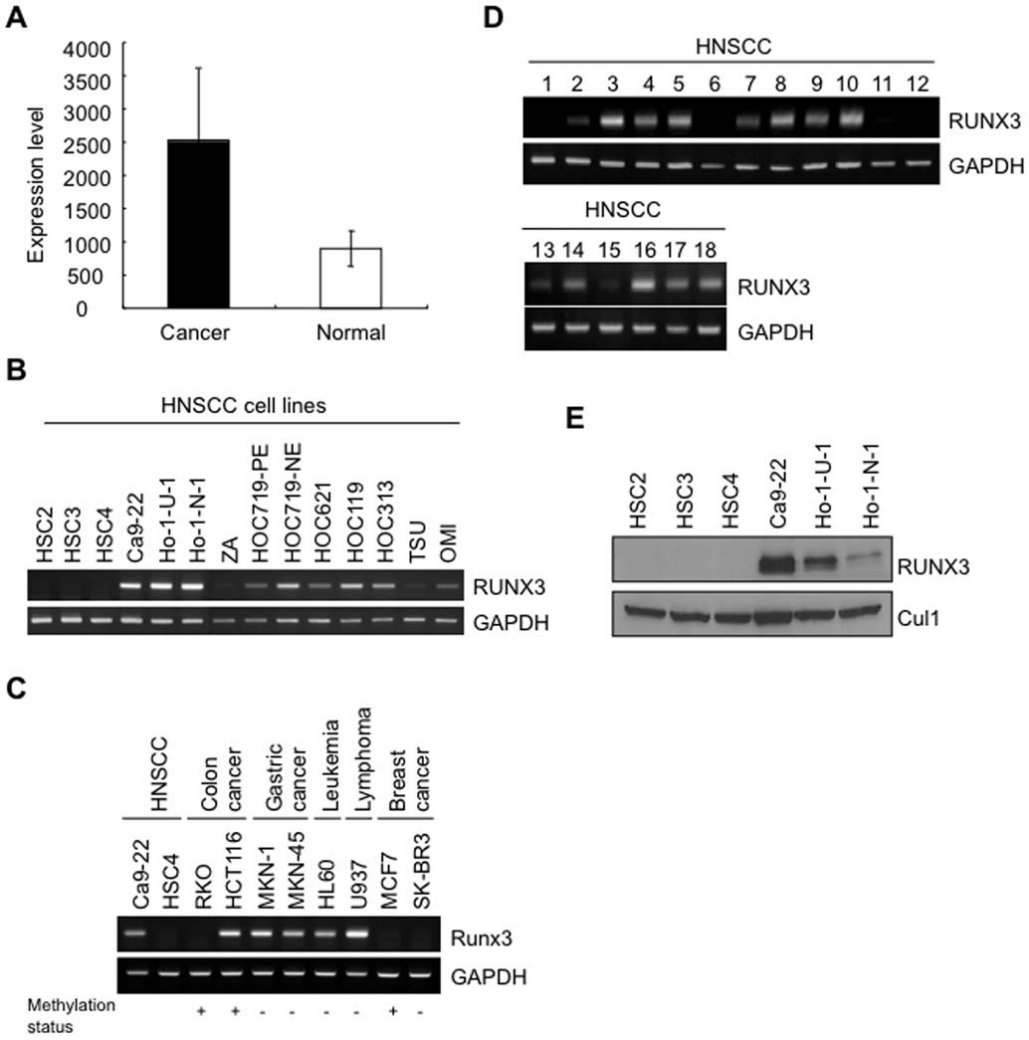
Expression of RUNX3 mRNA and protein in HNSCC. A: Total RNA from 41 primary HNSCC and 13 normal tissues was labeled and hybridized to Affymetrix U133A Gene Chips as previously reported. Graph shows the average of signal intensity of RUNX3 in 41 HNSCC and 13 normal tissues in microarray analysis. RUNX3 expression level in HNSCC is higher than that of normal tissues. B: Expression of RUNX3 mRNA in 14 HNSCC cell lines (HSC2, HSC3, HSC4, Ca9-22, Ho-1-N-1, Ho-1-U-1, ZA, HOC719-PE, HOC719-NE, HOC621, HOC119, HOC313, TSU and OMI) by RT-PCR. GAPDH was used as a control. C: Expression of RUNX3 mRNA in various cancer cell lines including colon cancer (RKO and HCT116), gastric cancer (MKN-1 and MKN-45), leukemia (HL60), lymphoma (U937) and breast cancer (MCF7 and SK-BR3). GAPDH was used as a control. D: Expression of RUNX3 mRNA in 18 HNSCC cases by RT-PCR. GAPDH was used as a control. E: Expression of RUNX3 protein in 6 HNSCC cell lines (HSC2, HSC3, HSC4, Ca9-22, Ho-1-N-1 and Ho-1-U-1) was examined by Western blot analysis. Cul1 expression was used as a loading control.

Head and neck squamous cell carcinoma (HNSCC) is one of the most common types of human cancer, with an annual incidence of more than 500,000 cases worldwide [10]. Like most epithelial cancers, HNSCC develops through the accumulation of multiple genetic and epigenetic alterations in a multi-step process [11]. Surprisingly, we found that RUNX3 expression level in HNSCC tissues was higher than that in normal tissues by a previously published microarray dataset of 41 HNSCC patients and 13 normal controls in our preliminary study [12] (Figure 1A). Interestingly, it recently has been reported that RUNX3 overexpression was observed in basal cell carcinoma of skin [13]. Therefore, we thought that RUNX3 might have an oncogenic role in HNSCC as well as in basal cell carcinoma of skin. In the present study, we examined the expression and roles of RUNX3 for HNSCC development.

## Materials and Methods

### Reagents

Transforming growth factor ß1 (TGF-ß), basic fibroblast growth factor (bFGF) and Platelet-derived growth factor-AA (PDGF-AA) were obtained from R&D systems (Minneapolis, MN). Insulin growth factor (IGF) was obtained from Sigma (Saint Louis, MO). Epidermal growth factor (EGF) was obtained from Pepro Tech EC (London, UK). Adriamycin (Doxorubicin hydrochloride) was obtained from Sigma.

### Cell lines and tissue samples

HNSCC cell lines (HSC2, HSC3, HSC4, Ca9-22, Ho-1-U-1 and Ho-1-N-1) were provided by Japanese Collection of Research Bioresources Cell Bank. HNSCC cell lines (ZA, HOC719-PE, HOC-719-NE, HOC621, HOC119, HOC313, TSU and OMI) were kindly provided by Dr. Kamata (Hiroshima University). Gastric cancer cell lines (MKN-1 and MKN-45), colon cancer cell lines (RKO and HCT116), breast cancer cell lines (MCF7 and SK-BR3), lymphoma cell line (U937) and leukemia cell line (HL-60) were provided by Japanese Collection of Research Bioresources Cell Bank. They were maintained in RPMI-1640 or Dulbecco's Modified Eagle Medium (Nissui Pharmaceutical Co., Tokyo, Japan) supplemented with 10% heat-inactivated FBS (Invitrogen) and 100 U/ml penicillin-streptomycin (Gibco) under conditions of 5% CO_2_ in air at 37°C. For growth assay, 5000 cells were plated onto 24 well plates (Falcon), and trypsinized cells counted at 0, 2, 4, 6 day by Cell Counter (Coulter Z1). Tongue tissues of mouse embryos at embryonic day 15.5 and 10 weeks old BALB/c mice were used for histology and immunohistochemistry. The experimental protocols were approved by the Animal Care and Use Committee of Hiroshima University.

Tissue samples of HNSCC were retrieved from the Surgical Pathology Registry of Hiroshima University Hospital, after approval by the Ethical Committee of Hiroshima University Hospital. Each patient gave written informed consent. Fifty-two cases of HNSCC and 9 normal oral mucosal tissues were used in this study. Five colon cancer tissues were also used for immunohistochemistry analysis (kindly provided by Dr. Shimamoto, Prefectural University of Hiroshima).

10% buffered-formalin fixed and paraffin embedded tissues were used for immunohistochemical examination. The histological grade and stage of tumor were classified according to the criteria of the Japan Society for Head and Neck Cancer. Fresh samples were taken from the HNSCC tissues for RT-PCR analysis.

### RT-PCR

Total RNA was isolated from cultures of confluent cells using the RNeasy Mini Kit (Qiagen). Preparations were quantified and their purity was determined by standard spectrophometric methods. cDNA was synthesized from 1 µg total RNA according to the ReverTra Dash (Toyobo Biochemicals, Tokyo, Japan). PCR amplification of RUNX3 was done using forward primer; 5′-CAGAAGCTGGAGGACCAGAC-3′ and reverse primer; 5′-TCGGAGAATGGGTTCAGTTC-3′. GAPDH was used as a control. Aliquots of total cDNA were amplified with Go Taq® Green Master Mix (Promega), and amplifications were performed in a PC701 thermal cycler (Astec, Fukuoka, Japan) for 30 cycles after an initial 30 sec denaturation at 94°C, annealed for 30 sec at 60°C, and extended for 1 min at 72°C in all primers. The amplification reaction products were resolved on 1.5% agarose/TAE gels (Nacalai tesque, Inc., Kyoto, Japan), electrophoresed at 100 mV, and visualized by ethidium-bromide staining.

### Western blot analysis

Western blotting was carried out as we described previously [14]. An anti-RUNX3 monoclonal antibody (R3-5G4, kindly provided by Dr. Ito, Institute of Molecular and Cell Biology, Singapore), anti-FLAG monoclonal antibody (M2, Sigma) and anti-Cul1 polyclonal antibody (Zymed) were used. Thirty µg of protein was subjected to 10% polyacrylamide gel electrophoresis followed by electroblotting onto a nitrocellulose filter. For detection of the immuno-complex, the ECL western blotting detection system (Amersham) was used.

### Immunohistochemical staining

Immunohistochemical detection of RUNX3 in HNSCC cases was performed on 4.5 µm sections mounted on silicon-coated glass slides, using a streptavidin-biotin peroxidase technique as described previously [14]. Immunohistochemical detection of Runx3 in human various cancer cases including 3 esophagus cancers, 3 gastric cancers, 3 colon cancers, 3 rectum cancers, 3 pancreas cancers, 3 liver cancers, 3 lung cancers, 3 kidney cancers, 3 bladder cancers, and 4 uterus cancers was performed using tissue microarray (MBL Co. Ltd., Nagoya, Japan). For immunohistochemical study, an anti-RUNX3 monoclonal antibody (R3-6E9, kindly provided by Dr. Ito, Institute of Molecular and Cell Biology, Singapore) was used. For immunohistochemical study of mouse tissues, an anti-Runx3 monoclonal antibody (R3-1E10, MBL Co. Ltd.) was used. The expression of RUNX3 was graded as positive (over 5% of tumor or epithelial cells showed immunopositivity) and negative (less than 5% of tumor or epithelial cells showed weak or focal immunopositivity or no staining). Three pathologists (Y.K., I.O., and T.T.) made all the assessments. Possible correlation between variables of the analyzed tumor samples was tested for association by the Fisher's exact test. A *P* value<0.05 was required for significance.

### 5-aza-2′-deoxycytidine treatment

HSC4 cells and normal epithelial cells were treated with medium containing 300 nM 5-aza-2′-deoxycytidine (5-aza-dC, Sigma) for 72 h. After treatment, cells were collected and examined the expression of RUNX3 mRNA by RT-PCR.

### Bisulfite modification and methylation-specific polymerase chain reaction (PCR)

**Figure 2:**
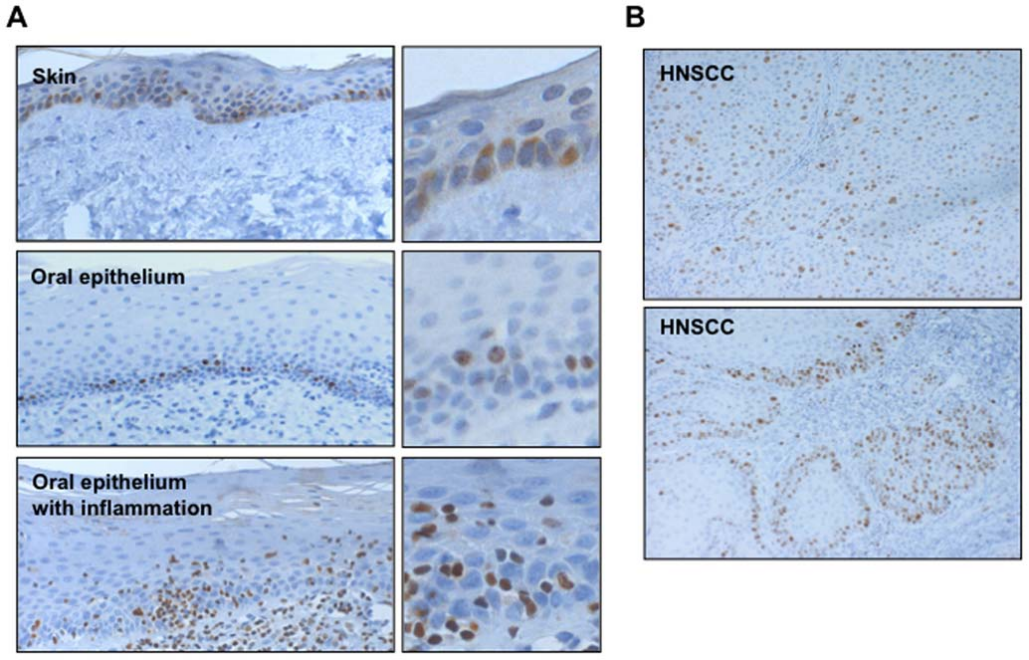
Immuno-expression of RUNX3 in HNSCC. A: Expression of RUNX3 was examined in skin epithelium (×100 and ×250, upper panel), normal oral mucosae (×100 and ×250, middle panel) and normal oral mucosae with infiltration of chronic inflammatory cells (×100 and ×250, lower panel). In skin epithelium, some of epidermal cells showed RUNX3 expression in its nuclei. In normal oral mucosal tissues, only a few epithelial cells in basal layer expressed RUNX3 in their nuclei, and most epithelial cells did not express RUNX3. In normal oral mucosae with infiltration of chronic inflammatory cells, lymphocytes infiltrating into oral mucosa showed RUNX3 expression. B: Expression of RUNX3 was examined in HNSCC cases (×100). Most cancer cells expressed RUNX3 in their nuclei.

Genomic DNA from cells or tissues was extracted using the DNeasy Kit (Qiagen, Hilden, Germany). Fifty micro liters of the supernatant were used directly as a source of DNA for sodium bisulfite treatment. DNA samples were treated with bisulfite to convert all unmethylated cytosines to uracils whilst leaving methylated cytosines unaffected. DNA was denatured by NaOH (final concentration, 0.2 M) for 10 min at 37°C. Thirty µl of 10 mM hydroquinone (Sigma) and 520 µl of 3 M sodium bisulfite (Sigma) at pH 5.0 both freshly prepared, were added and mixed, and samples were incubated at 50°C for 16 h. Modified DNA was purified using Wizard DNA purification resin (Promega) and eluted into 50 µl of water. Modification was completed by NaOH (final concentration, 0.3 M) treatment for 5 min at room temperature, followed by ethanol precipitation. Modified DNAs were amplified in a 20 µL reaction volume containing 2 µL 10×PCR buffer with 15 mM MgCl2, 4 µL 5xQ-Solution, 10 pM of each primer, 0.2 mM dNTPs and 0.75 U Taq polymerase (HotStar Taq DNA polymerase; Qiagen, Hilden, Germany). After the mixture was heated at 95°C for 15 min, PCR was performed in a thermal cycler (GeneAmp 2400; PE Applied Biosystems, Foster City, CA, USA) for 35 cycles of denaturation at 94°C for 30 sec, annealing at 58°C for 60 sec, and extension at 72°C for 60 sec, followed by a final 10 min extension at 72°C. The PCR products were separated on a 8% non-denaturing polyacrylamide gel. RUNX3 CpG island and analyzed regions (No. 1–10) are shown in Figure 2B. The primer sets used in this study were listed in Table S1 (GenBank accession number AL023096) [15].

### Generation of RUNX3-overexpressing HNSCC cells

A RUNX3 expression plasmid, pcDNA3 encoding FLAG tagged RUNX3 cDNA, was kindly provided by Dr. Ito (Institute of Molecular and Cell Biology, Singapore). The RUNX3/pcDNA3 plasmid or the vector alone was introduced into HSC3 cells, and then G418 (500 µg/ml, Gibco) was added to the culture medium after 48 h of transfection. After 2 weeks of G418 selection, we obtained the stable pool clones. Cell transfections were performed using FuGENE 6 (Roche) according to the manufacture's instruction.

### Silencing by Small Interfering RNA

Logarithmically growing Ca9-22 cells were seeded at a density of 10^5^cells/6 cm dish and transfected with oligos twice (at 24 and 48 h after replating) using Oligofectamine (Invitrogen) as described [16]. Forty-eight hours after the last transfection, lysates were prepared and analyzed by SDS-PAGE and immunoblotting. The siRNA is a 19-bp duplex oligoribonucleotide with a sense strand corresponding to nucleotides 275–293 of human RUNX3 mRNA sequence; 5′-ccuucaaggugguggcauu-3′. A scrambled sequence that does not show significant homology to rat, mouse or human gene sequences was used as a control.

### Flow cytometric analysis

Cell cycle distribution was determined by DNA content analysis after propidium iodide staining. Cells were cultured as described above, and fixed in 70% ethanol and stored at 4°C before analysis. Flow cytometric determination of DNA content was analyzed by a FACS calibur (Becton-Dickinson, San Jose, CA) flow cytometer. For each sample, 20,000 events were stored.

### Annexin V and Propidium Iodide Dual-Staining Assay

After serum starvation or Dox treatment, the cells were then stained with fluorescein isothiocyanate (FITC)-conjugated Annexin V and propidium iodide (PI), using the Annexin V-FITC Apoptosis Detection kit (Becton-Dickinson) according to the manufacturer's instructions. Apoptotic cells were identified by dual-staining with recombinant FITC-conjugated with Annexin V and propidium iodide (PI). Data acquisition and analysis were done in a FACSCalibur (Becton-Dickinson) flow cytometer using CellQuest software.

## Results

### Highly expression of RUNX3 in HNSCC

First, we examined the expression of RUNX3 in 14 HNSCC cell lines (HSC2, HSC3, HSC4, Ca9-22, Ho-1-U-1, Ho-1-N-1, ZA, HOC719-PE, HOC719-NE, HOC621, HOC119, HOC313, TSU, and OMI). RUNX3 mRNA was observed in 9 of 14 HNSCC cell lines (Figure 1B). In certain type of human cancers including gastric and bladder, it has been reported that point mutations of RUNX3 were observed [3], [5]. However, no mutations were found in HNSCC cell lines with RUNX3 mRNA expression (Ca9-22, Ho-1-U-1 and Ho-1-N-1) (data not shown). RUNX3 mRNA expression was also examined in various cancer cell lines including colon cancer (RKO and HCT116), gastric cancer (MKN-1 and MKN-45), leukemia (HL60), lymphoma (U937) and breast cancer (MCF7 and SK-BR3) (Figure 1C). RKO, MCF7 and SK-BR3 cells did not express RUNX3 mRNA. As previously reported, RUNX3 expression was well correlated with methylation status, except for SK-BR3 cells [17]–[20]. In SK-BR3 cells, down-regulation of RUNX3 is thought to be caused by unknown mechanism [20]. We also examined the expression of RUNX3 mRNA in 18 HNSCC tissues. In 13 of 18 (72.2%) HNSCC tissues, RUNX3 mRNA expression was observed (Figure 1D). Next, RUNX3 protein expression was examined by Western blot analysis in 6 HNSCC cells (HSC2, HSC3, HSC4, Ca9-22, Ho-1-U-1 and Ho-1-N-1) (Figure 1E). Among them, Ca9-22, Ho-1-U-1 and Ho-1-N-1 cells expressed RUNX3 protein, corresponding to mRNA expression. Then, we examined RUNX3 expression in 9 normal oral mucosal tissues and 52 HNSCC cases by immunohistochemistry. First, we used skin epithelium and colon mucosa for reactivity of RUNX3 expression. As shown in recent report [13], epidermal cells also showed RUNX3 expression in its nuclei (Figure 2A, upper panel). We also examined the RUNX3 expression in colon cancer. In colon cancer, RUNX3 is thought to be a tumor suppressor gene [18]. As previously reported [18], RUNX3 expression was observed in its nuclei of normal mucosa, while colon cancer cells did not express RUNX3 (Figure S1). In normal oral mucosal tissues, only a few epithelial cells in basal layer slightly expressed RUNX3 in their nuclei, and most epithelial cells did not express (Figure 2A, middle panel). Lymphocytes infiltrating into normal oral mucosa showed RUNX3 expression (Figure 2A, lower panel). Immunopositivity of RUNX3 in lymphocytes was used as a positive control of staining. On the other hand, most cancer cells expressed RUNX3 in their nuclei (Figure 2B). We also confirmed that nuclear expression of RUNX3 by using nuclear and cytoplasmic fraction of HNSCC cell lines, indicating no protein mislocalization (data not shown). RUNX3 expression was frequently observed in 50% (26 of 52) of HNSCC cases (Table 1). Moreover, we compared RUNX3 expression with clinico-pathological findings including differentiation, invasiveness and metastasis. Interestingly, RUNX3 expression was well correlated with malignant behaviors including poorly differentiation, invasiveness and metastasis (Table 1).

**Table 1:** Runx3 expression in normal oral mucosa and HNSCC.

			Total	Runx3 expression Negative*	Runx3 expression Positive*	*P* value
Normal			9	9 (100%)	0 (0%)	0.005
HNSCC			52	26 (50%)	26 (50%)	
HNSCC	Histology	Well	31	18 (58%)	13 (42%)	0.031
		Moderate	13	8 (62%)	5 (38%)	
		Poorly	8	0 (0%)	8 (100%)	
	Invasion**	I & II	26	18 (69%)	8 (31%)	0.006
		III & IV	26	8 (31%)	18 (69%)	
	Metastasis	Negative	38	24 (63%)	14 (37%)	0.005
		Positive	14	2 (14%)	12 (86%)	

* Runx3 expression is graded as negative (less than 5% of positive cells) and positive (over 5% of positive cells).

** The grading of invasion is according to the classification described by Jakobsson et al. as patterns I, II, III and IV [38].

### Methylation status of *RUNX3* was well correlated with its mRNA expression in HNSCC

**Figure 3:**
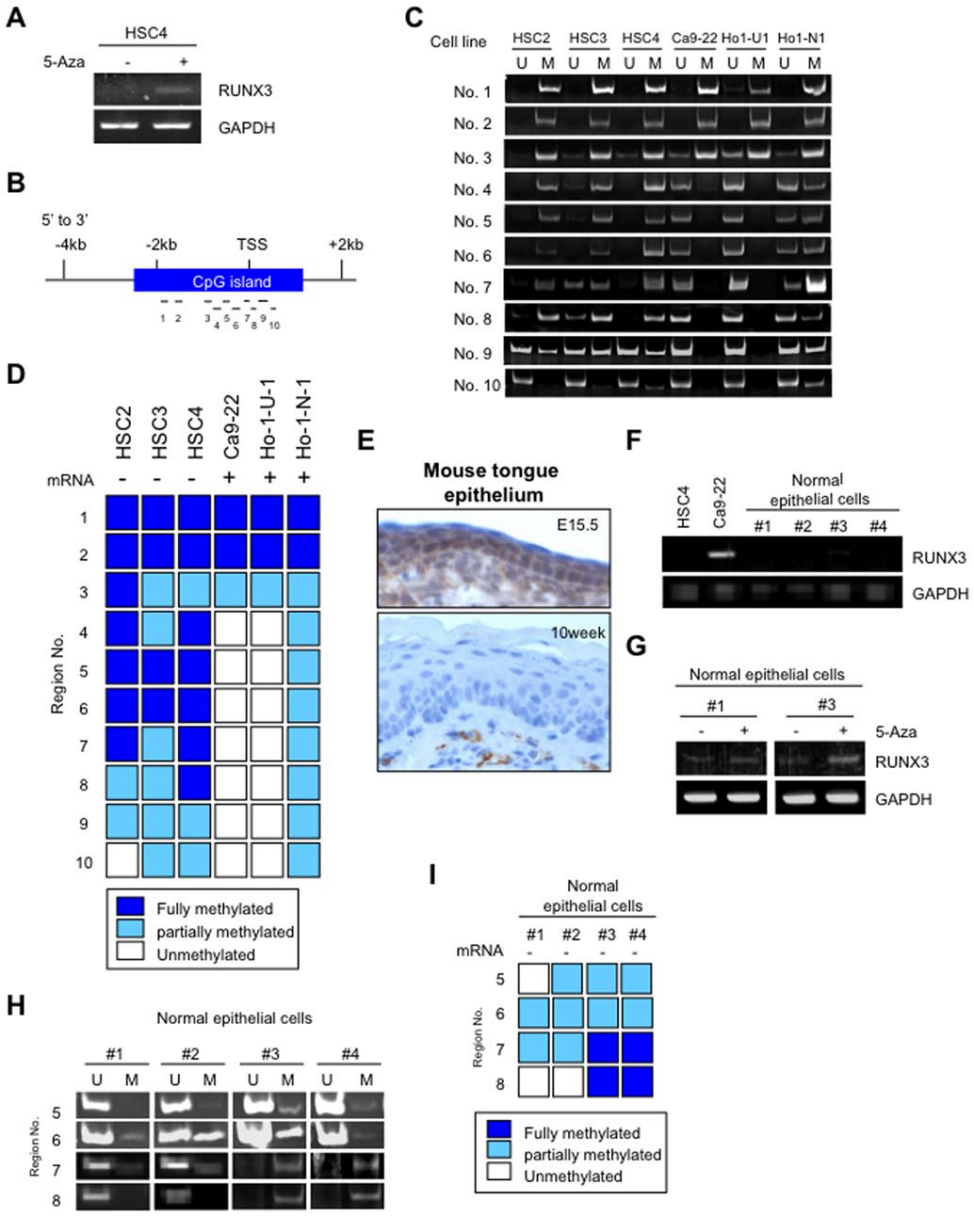
Methylation status in normal oral epithelial cells and HNSCC. A: RUNX3 expression was examined after 5-aza-2′-deoxycytidine (5-Aza) treatment. HSC4 cells were treated with medium containing 300 nM 5-aza-2′-deoxycytidine for 72 h. After treatment, cells were collected and examined the expression of RUNX3 mRNA by RT-PCR. B: *RUNX3* CpG island and analyzed regions (No. 1–10) are shown as vertical bars. Transcriptional start site (TSS) is located within region No. 7. C: Methylation status of RUNX3 in HNSCC cell lines. Genomic DNA was extracted from HNSCC cell lines and was treated with bisulfite. Methylation status of promoter region (No. 1–10) was examined by methylation specific PCR method. D: The summary of methylation and expression status of RUNX3 in HNSCC cell lines. E: Expression of Runx3 in mouse tongue epithelium of embryo (upper panel) and adult (lower panel) mouse by immunohistochemistry. Tongue tissues of mouse embryos at embryonic day 15.5 and 10 weeks old BALB/c mice were used. F: RUNX3 mRNA expression was examined by RT-PCR in 4 primary cultured normal oral epithelial cells (#1–#4). HSC4 cell was used as a negative control and Ca9-22 cell was used as a positive control for RUNX3 expression. GAPDH was used as a control. G: RUNX3 expression was examined after 5-aza-2′-deoxycytidine (5-Aza) treatment. Primary cultured normal oral epithelial cells (#1 and #2) were treated with medium containing 300 nM 5-aza-2′-deoxycytidine for 72 h. After treatment, cells were collected and examined the expression of RUNX3 mRNA by RT-PCR. H: Genomic DNA was extracted from 4 primary cultured normal oral epithelial cells (#1–#4). Methylation status at region No. 5–8 was examined by methylation specific PCR method. I: The summary of methylation and expression status of RUNX3 in normal epithelial cells.

We asked how RUNX3 expression was caused by in HNSCC. Reduced expression of RUNX3 was frequently caused by CpG island hypermethylation in various types of cancer [9]. In fact, RUNX3 expression was observed in HSC4 cells without RUNX3 expression after 5-aza-2′-deoxycytidine treatment (Figure 3A). Homma et al. examined the methylation status of multiple regions of *RUNX3* promoter CpG island (3478 bp) within the proximal promoter (No. 1–10) in gastric cancers and found that methylation at the region spanning the transcription start site (No. 5–8) is critical for loss of the RUNX3 expression (Figure 3B) [15]. To examine the *RUNX3* methylation status in HNSCC, we analyzed the methylation of *RUNX3* at all promoter regions (No. 1–10) by methylation-specific PCR on the panel of HNSCC cell lines (Figure 3C and 3D). HNSCC cells with RUNX3 expression showed unmethylation or partially methylation at No. 5–8 region (Figure 3D). HNSCC cells without RUNX3 expression showed fully methylation at No. 5 and No. 6 region. Thus, methylation status of the *RUNX3* promoter region was well correlated with RUNX3 mRNA expression in HNSCC cell lines.

Recent report shows that Runx3 is expressed in the tongue and palate epithelium of mouse embryos from embryonic day 12.5 to 16.5, and that Runx3 expression decreases after embryonic day 16.5 and disappears in newborn mice [21]. Here we also confirmed that Runx3 expression was observed in tongue epithelium of mouse embryos (E15.5), but not in adult mouse tongue epithelium (10 weeks old) (Figure 3E). These findings made us hypothesize that *RUNX3* might be silenced by methylation in adult oral epithelial cells. Oral mucosal tissue usually contains connective tissue and infiltrating lymphocytes. As shown in Figure 2A (lower panel), lymphocytes infiltrating into normal oral mucosa showed RUNX3 expression. Therefore, we used primary cultured epithelial cells obtained from normal oral epithelium in this study for avoiding contamination of lymphocytes. In similar to immunohistochemical studies (Figure 2A), 4 primary cultured epithelial cells obtained from normal oral epithelium (#1–#4) did not express RUNX3 mRNA (Figure 3F), indicating that expression level of RUNX3 is low or absent in adult normal oral epithelium. We examined the RUNX3 expression after 5-aza-2′-deoxycytidine treatment. RUNX3 expression was up-regulated by 5-aza-2′-deoxycytidine treatment in 2 primary cultured epithelial cells (#1 and #3) (Figure 3G). Then, we examined whether *RUNX3* was silenced by methylation in adult oral epithelial cells. We performed methylation-specific PCR in 4 primary cultured epithelial cells at the region spanning the transcription start site of *RUNX3* (No. 5–8), which is critical for loss of the RUNX3 expression in HNSCC cells (Figure 3C). Interestingly, 2 primary cultured epithelial cells (#1 and #2) showed partially methylation at No. 6 and No. 7 region, and another 2 primary cultured epithelial cells (#3 and #4) showed fully methylation at No. 7 and No. 8 region (Figure 3H and 3I).

### RUNX3 overexpression enhanced cell proliferation and inhibited apoptosis

**Figure 4:**
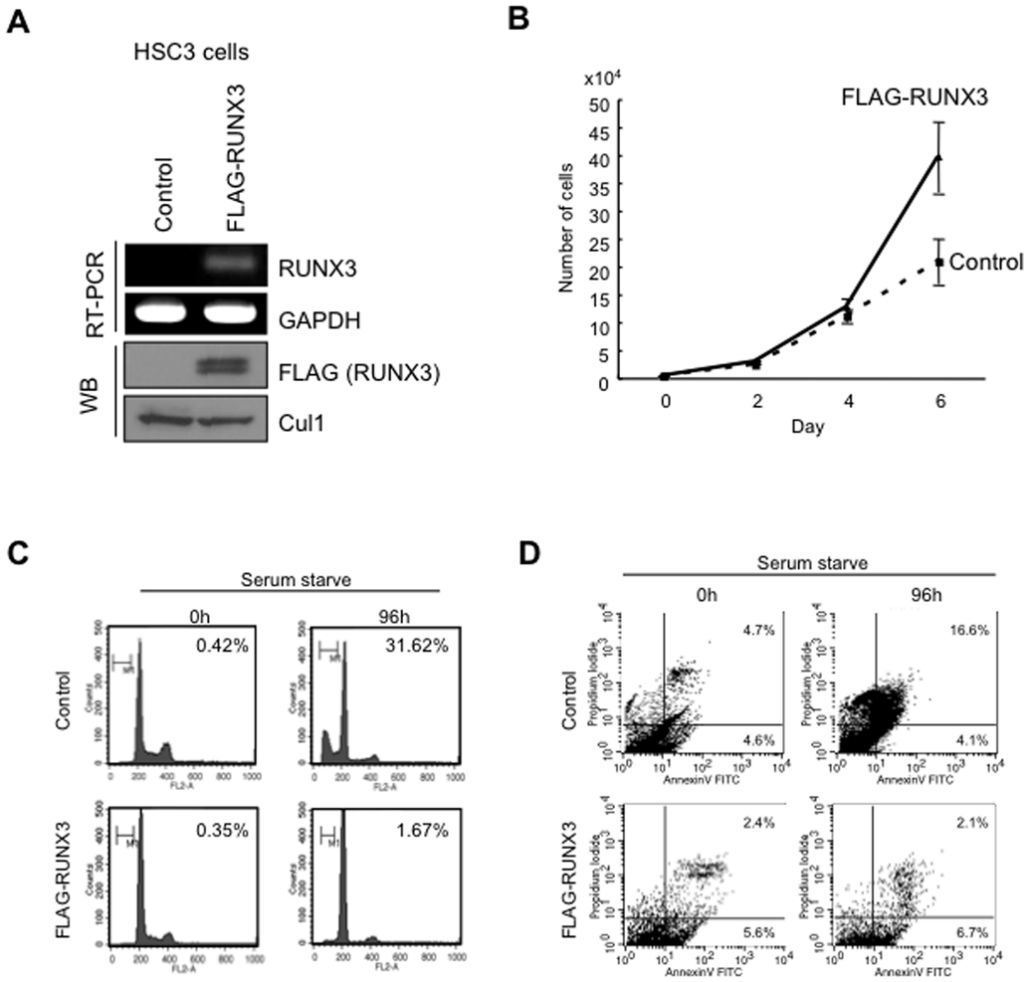
RUNX3 overexpression promoted cell growth and inhibited serum starvation induced apoptosis. A: Generation of RUNX3 overexpressing cells. The RUNX3/pcDNA3 plasmid or the vector alone was introduced into HSC3 cells, and the stable pool clones were obtained by G418 selection for 2 weeks. Exogenous expression of RUNX3 mRNA and protein was examined by RT-PCR and Western blot analysis (WB). Cul1 was used as a loading control. B: The graph shows cell growth of RUNX3 overexpressing and control HSC3 cells. Cells were plated on 24 well plates, and trypsinized cells were counted by Cell Counter (Coulter Z1) at 0, 2, 4 and 6 day. C: RUNX3 overexpression inhibited the serum starvation induced apoptosis. Cells were incubated for 0, 48 and 96 hours after serum starvation and fixed in 70% ethanol. Cell cycle distribution was determined by DNA content analysis after propidium iodide staining using a flow cytometer. For each sample, 20,000 events were stored. We performed two independent experiments. D: Flow cytometric analysis of Annexin V and propidium iodide staining in control and RUNX3 overexpressing cells after serum starvation for 96 h. We performed two independent experiments.

**Figure 5 pone-0005892-g005:**
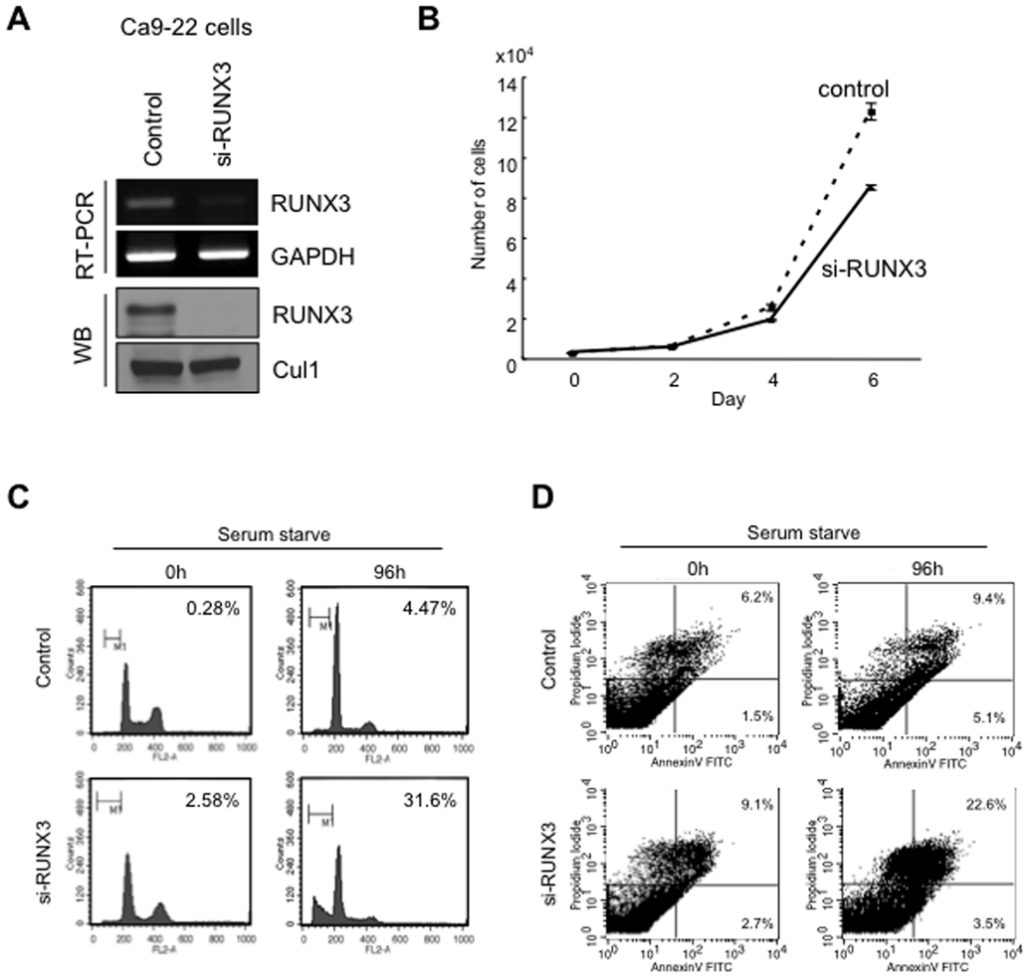
RUNX3 knockdown inhibited cell growth and enhanced serum starvation induced apoptosis. A: RUNX3 silencing by small interfering RNA. Logarithmically growing Ca9-22 cells with RUNX3 expression were seeded at a density of 10^5^cells/6 cm dish and transfected with oligos twice (at 24 and 48 hr after replating). Forty-eight hours after the last transfection, cells were collected and RUNX3 mRNA and protein expression was examined by RT-PCR and Western blot analysis. B: The graph shows that cell growth of RUNX3 siRNA treated and control Ca9-22 cells. Cells were plated on 24 well plates, and trypsinized cells were counted by Cell Counter at 0, 2, 4 and 6 day. C: RUNX3 siRNA enhanced the serum starvation induced apoptosis. Cell cycle distribution was determined by DNA content analysis after propidium iodide staining using a flow cytometer. For each sample, 20,000 events were stored. We performed two independent experiments. D: Flow cytometric analysis of Annexin V and propidium iodide staining in control and RUNX3 knockdown cells after serum starvation for 96 h. We performed two independent experiments.

To know the role of RUNX3 in HNSCC, we stably transfected an expression vector of FLAG-RUNX3 into HSC3 cells with lower expression of RUNX3. We obtained stably RUNX3 overexpressing cells (Figure 4A). Interestingly, RUNX3 overexpression enhanced cell growth (Figure 4B). At 6day, number of RUNX3 overexpressing cells was remarkably higher than that of control cells. Although we examined migration and invasion, RUNX3 overexpression did not promote migration and invasion (Figure S2). Moreover, increased population corresponding to sub-G1 after serum starvation was observed only in control cells, but not in RUNX3 overexpressing cells (Figure 4C). To demonstrate whether this increment of sub-G1 detected in control cells after serum starvation is due to apoptosis, the level of apoptosis was assessed by using annexin V-FITC/propidium iodide assays (Figure 4D). Annexin V/propidium iodide double-positive cells were observed in 1.8% of RUNX3 overexpressing cells, while in 21.7% of control cells, indicating that RUNX3 overexpression inhibited serum starvation induced apoptosis. To confirm these phenotypes, we examined the knockdown of RUNX3 in Ca9-22 cells, which showed RUNX3 overexpression and were resistant to serum starvation-induced apoptosis. For knockdown of RUNX3, we used three different siRNAs (si-RUNX3-1, si-RUNX3-2 and si-RUNX3-3) and their cocktail. RUNX3 expression was remarkably silenced by si-RUNX3-1 (Figure S3A). Therefore, we used si-RUNX3-1 for the following studies. RUNX3 siRNA transfection reduced the expression of RUNX3 mRNA and protein in Ca9-22 cells (Figure 5A). We examined if siRNA induced a non-specific interferon stress response. RUNX3 siRNA treatment did not induce classic interferon-responsive genes, OAS1 and ISG54 mRNAs, indicating that RUNX3 siRNA treatment did not induce significant interferon response (Figure S3B). As we expected, RUNX3 siRNA inhibited the cell growth (Figure 5B). At 6day, number of RUNX3 siRNA treated cells was remarkably lower than that of control cells. Moreover, RUNX3 siRNA increased the population corresponding to sub-G1 after serum starvation (Figure 5C). Annexin V/propidium iodide double-positive cells were observed in 10.5% of control cells, while in 18.1% of RUNX3 knockdown cells (Figure 5D).

**Figure 6:**
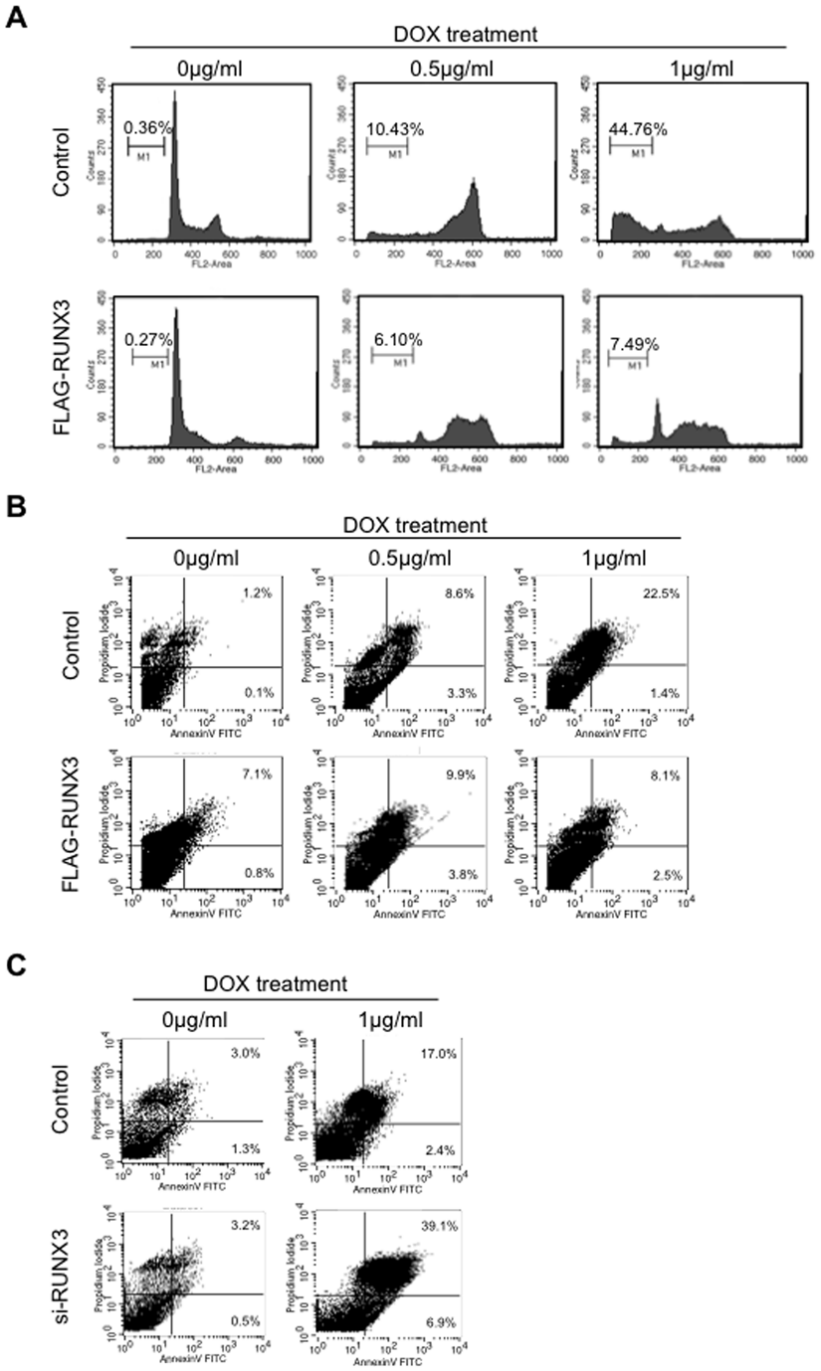
RUNX3 overexpression inhibited chemotherpeutic drug induced apoptosis. A: Adriamycin (Dox, 0, 0.5 and 1 µg/ml) was treated for 72 hours in control and RUNX3 overexpressing HSC3 cells. Cell cycle distribution was determined by DNA content analysis after propidium iodide staining using a flow cytometer. For each sample, 20,000 events were stored. Percentage of sub-G1 population is indicated. We performed two independent experiments with triplicate wells per condition. Representative data is shown. B: Flow cytometric analysis of Annexin V and propidium iodide staining in control and RUNX3 overexpressing cells after DOX treatment for 72 h at the indicated doses. C: Flow cytometric analysis of Annexin V and propidium iodide staining in control and RUNX3 knockdown cells after DOX treatment for 72 h at the indicated doses. We performed two independent experiments.

In addition, we examined the inhibition of apoptosis after treatment with chemotherapeutic drug in control and RUNX3 overexpressing HNSCC (Figure 6). Control and RUNX3 overexpressing HSC3 cells were exposed for 72 hours to adriamycin (DOX; 0.5 and 1 µg/ml), which is a chemotherapeutic agent commonly used in the treatment of HNSCC. The sub-G1 population of RUNX3 overexpressing HSC3 cells was 7.49%, while that of control cells was 44.76% after treatment with 1 µg/ml of DOX (Figure 6A). Moreover, after treatment with 1 µg/ml of DOX, Annexin V/propidium iodide double-positive cells were observed in 25.2% of control cells, while in 8.9% of RUNX3 overexpressing cells (Figure 6B). On the other hand, Annexin V/propidium iodide double-positive cells increased by RUNX3 knockdown (Figure 6C). Overall these suggest that RUNX3 overexpression may be involved in HNSCC development through promoting cell growth and inhibition of apoptosis.

To know the role of RUNX3 in tumorigenesis, we examined the tumorsphere formation by using ultra low attachment plates. RUNX3 overexpression promoted tumorsphere formation (Figure S4A and S4B). The average number of colonies was 535.6 and 663.3 in control and RUNX3 overexpressing cells, respectively (Figure S4B). Moreover, RUNX3 knockdown inhibited tumorsphere formation (Figure S4C and S4D). The average number of colonies was 566.0 and 161.3 in control and RUNX3 knockdown cells, respectively (Figure S4D).

Moreover, we compared the gene transcriptional profiles between control and RUNX3 overexpressing HSC3 cells. By microarray analysis, 1251 genes were selectively up-regulated (over 2-fold) and 472 genes were down-regulated (less than 0.5) in RUNX3 overexpressing HSC3 cells (Dataset S1). Table S2 shows the list of up-regulated genes (over 15-fold) and down-regulated genes (less than 0.1) in RUNX3 overexpressing HSC3 cells, respectively. Then, we used the computer program Onto-Express to analyze the identified oxidized mRNA species and grouped them according to cellular component or molecular function [22]. As shown in Figure S5A, 1251 genes that were up-regulated by RUNX3 overexpression in HNSCC cells were classified into gene ontology categories according to involvement in different biological processes. Interestingly, up-regulated genes by RUNX3 overexpression belong to apoptosis, cell cycle and signal transduction (Figure S5A and S5B). Moreover, some genes were classified as being involved in keratinocyte differentiation, keratinocyte proliferation and response to serum starvation (Figure S5A and S5B). Thus, these focused genes potentially are important for apoptosis, cell growth and cellular differentiation by RUNX3 overexpression. RUNX3 is known as a nuclear effecter of the TGF-ß/BMP pathways, and a key tumor suppressor gene in the gastric epithelium [2], [3]. In vertebrate facial development, BMPs, TGF-ß, Shh and FGFs are known to be involved [23]. In addition, Runx3 is expressed in the tongue and palate epithelium of mouse embryos [20]. Therefore, we suggest that RUNX3 may be involved in the development of oral mucosa through growth factor signaling pathways. HNSCC cell line, HOC621 cells with lower expression of RUNX3 mRNA were treated with growth factors including TGF-ß1, IGF, EGF, bFGF and PDGF-AA (Figure S6A). Among these growth factors, EGF significantly enhanced RUNX3 expression in HOC621 cells. EGF enhanced RUNX3 expression in concentration and time dependent manner (Figure S6B). However, RUNX3 expression was not induced by growth factor stimulation in other HNSCC cells without RUNX3 expression due to methylation (data not shown). Moreover, we examined the expression of EGF and EGFR and compared with RUNX3 expression. Interestingly, EGF expression was well correlated with RUNX3 expression (Figure S6C).

## Discussion

RUNX3 is inactivated in more than 80% of human gastric cancer by CpG island hypermethylation, protein mislocalization and point mutations [3]–[5]. Moreover, it has been reported that reduced expression of RUNX3 was observed in various cancers including bladder, liver, colon and lung cancers [5]–[8]. In fact, gastric mucosa of Runx3-null mice develops hyperplasia due to the stimulated proliferation and suppressed apoptosis of epithelial cells [3]. Taken together, RUNX3 acts as a tumor suppressor in various cancers. In this study, we confirmed the low expression of RUNX3 in various cancer cell lines by RT-PCR (Figure 1C). Expression level of RUNX3 was well correlated with methylation status, as previously reported [17]–[20]. By immunohistochemistry, RUNX3 expression was observed in normal colon mucosa, while cancer cells did not express RUNX3 (Figure S1). Contrary to these tumors, here we found oncogenic roles for RUNX3 in HNSCC. It recently has been shown that RUNX3 overexpression was observed in basal cell carcinoma of skin [13]. This distinct role in HNSCC and skin basal cell carcinoma may be accounted for by the pathogenesis of both types of cancer, which arise from squamous epithelium. In fact, RUNX3 frequency of RUNX3 positive cells is low in adult skin and oral mucosa, while most cancer cells expressed (Figure 2) [13]. Overexpression of RUNX3 has previously been associated with functional mutations or protein mislocalization in breast, gastric and bladder tumors [4], [5]. However, functional mutations or protein mislocalization were not found in HNSCC, indicating that RUNX3 is fully functional in HNSCC cells. All members of the RUNX family, most notably Runx2, are known to promote tumorigenecity in mouse models [24], [25]. In fact, RUNX3 overexpression promoted cell proliferation. In Figure 4B and 5B, RUNX3 overexpression or knockdown affected to the cell growth from 4 day after plating. Therefore, RUNX3 may be involved in allowance for high cell density in addition to stimulation of cell growth. Moreover, RUNX3 overexpression inhibited serum starvation induced apoptosis and chemotherapeutic drug induced apoptosis *in vitro* in HNSCC cells (Figures 4–6). Interestingly, RUNX3 overexpression promoted tumorigenesis, demonstrated by tumorsphere formation assay (Figure S4). These findings may be implicated in RUNX3 related malignant behaviors of HNSCC including invasiveness and metastasis (Table 1). Comparing the gene expression profile between control and RUNX3 overexpressing HNSCC cells revealed that several genes were selectively up-regulated and down-regulated (Table S2). CXCL14, IGFBP2 and EphA4 receptor were up-regulated, and fibronectin 1 was down-regulated in RUNX3 overexpressing HNSCC cells. Although CXCL14 suppresses tumor growth in some type of cancer, CXCL14 is involved in invasion of pancreatic cancer [26]. Moreover, IGFBP-2 is a highly sensitive marker of malignant progression in different tumors and potentially involved in anti-apoptosis, angiogenesis, and metastasis during cancer progression [27]. EphA4 receptor also promotes cancer cell growth [28]. In addition, classification into gene ontology categories according to involvement in different biological processes showed that several genes involved in apoptosis and cell cycle were up-regulated by RUNX3 overexpression (Figure S5). It is interesting to examine the correlation between RUNX3 and these molecules. The findings identified by microarray analysis may help future experiments for clarifying the detailed role of RUNX3 in the development of HNSCC.

**Figure 7:**
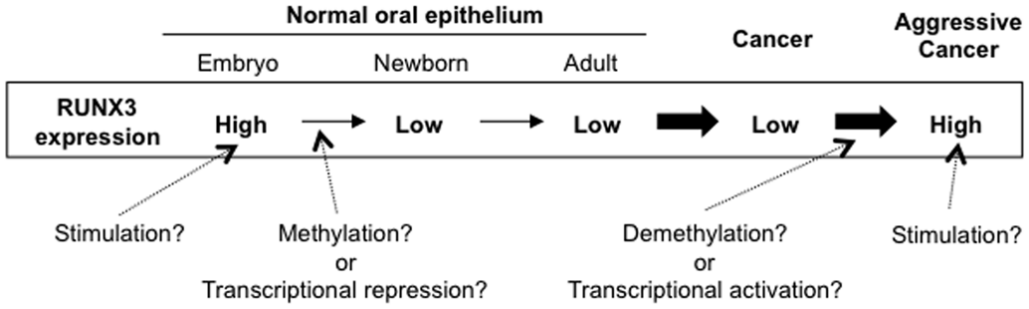
Schematic model of RUNX3 overexpression in HNSCC. In oral epithelium of embryo, RUNX3 is expressed and may be induced by some signaling pathways such as BMP/TGF-ß, Shh and FGF. After birth, RUNX3 may be methylated of its promoter and/or transcriptional repression in oral epithelial cells. RUNX3 may be caused by demethylation or transcriptional modulation during cancer development. In aggressive HNSCC, unknown stimulation may enhance RUNX3 expression.

DNA methylation plays an important role in the establishment and maintenance of the program of gene expression. The pattern of 5-methylcytosine distribution in the genome is unique for each cell type and is established in embryogenesis as a result of balance between DNA methylation and demethylation [29], [30]. It recently has been revealed that Runx3 is expressed in the tongue and palate epithelium of mouse embryos and Runx3 expression disappears in newborn mice [20]. Here, we also confirmed that Runx3 expression was observed in tongue epithelium of embryo, but not in adult tongue epithelium (Figure 3E). Interestingly, we demonstrated that *RUNX3* methylation of its promoter region was observed in normal epithelial cells (Figure 3H and 3I), suggesting that RUNX3 may be silenced by methylation in adult normal oral epithelium. However, there may be another possibility, such as transcriptional modulation by traditional activators or repressors, in addition to the regulation of RUNX3 expression by methylation. Moreover, methylation of its promoter region was observed in HNSCC cells without RUNX3 expression (Figure 3C and 3D). Therefore, these findings indicate that RUNX3 expression in HNSCC may be caused by demethylation during cancer development (Figure 7). In malignant tumor, the level of general demethylation and the frequency of demethylation increase with tumor progression [31], [32], and demethylation of individual CpG dinucleotides located in C-MYC, Ha RAS, and ERB-A1 proto-oncogene was revealed in human tumors [33]–[36]. Detailed mechanism of RUNX3 regulation in normal adult oral epithelium and HNSCC requires further experiments.

RUNX3 is expressed in the gastrointestinal tract of mouse and human [3], and RNT, a homolog of the RUNX family in C. elegans, is involved in the development of a functional epidermis and gut [37]. RUNX3 is known as a nuclear effector of the functional BMP/TGF-ß pathways, and a key tumor suppressor gene in the gastric epithelium [2], [3]. In vertebrate facial development, BMPs, TGF-ß, Shh and FGFs are known to be involved [23]. As RUNX3 expression in tongue and palatal epithelium is observed only in embryonic stage, RUNX3 may be involved in the development of oral mucosa through some signaling pathways such as TGF-ß/BMP, Shh and FGF. We treated growth factors such as TGF-ß1, IGF, EGF, bFGF, and PDGF-AA in HNSCC cells with RUNX3 expression and found that EGF enhanced RUNX3 expression. In HNSCC, RUNX3 overexpression may be caused by some stimulation including EGF (Figure 7). To know the mechanism of RUNX3 overexpression in HNSCC requires further studies.

Overall we suggest that i) RUNX3 has an oncogenic role in HNSCC, ii) RUNX3 expression observed in HNSCC may be caused in part by demethylation during cancer development, and iii) RUNX3 expression can be a useful marker for predicting malignant behavior and the effect of chemotherapeutic drugs in HNSCC.

## Supporting Information

Table S1 (0.05 MB PPT)

Table S2 (0.12 MB DOC)

Figure S1 (0.35 MB TIF)

Figure S2 (0.39 MB TIF)

Figure S3 (0.17 MB TIF)

Figure S4 (0.31 MB TIF)

Figure S5 (0.90 MB TIF)

Figure S6 (0.20 MB TIF)

Dataset S1 (0.88 MB XLS)
