# Supplementary material for: RUNX3 Has an Oncogenic Role in Head and Neck Cancer
Source: PLoS One. 2009 Jun 12;4(6):e5892. doi: 10.1371/journal.pone.0005892 (PMC2690822; doi:10.1371/journal.pone.0005892)
Supplement: Table S1 — (0.05 MB PPT) [file pone.0005892.s001.ppt]

## Slide 1
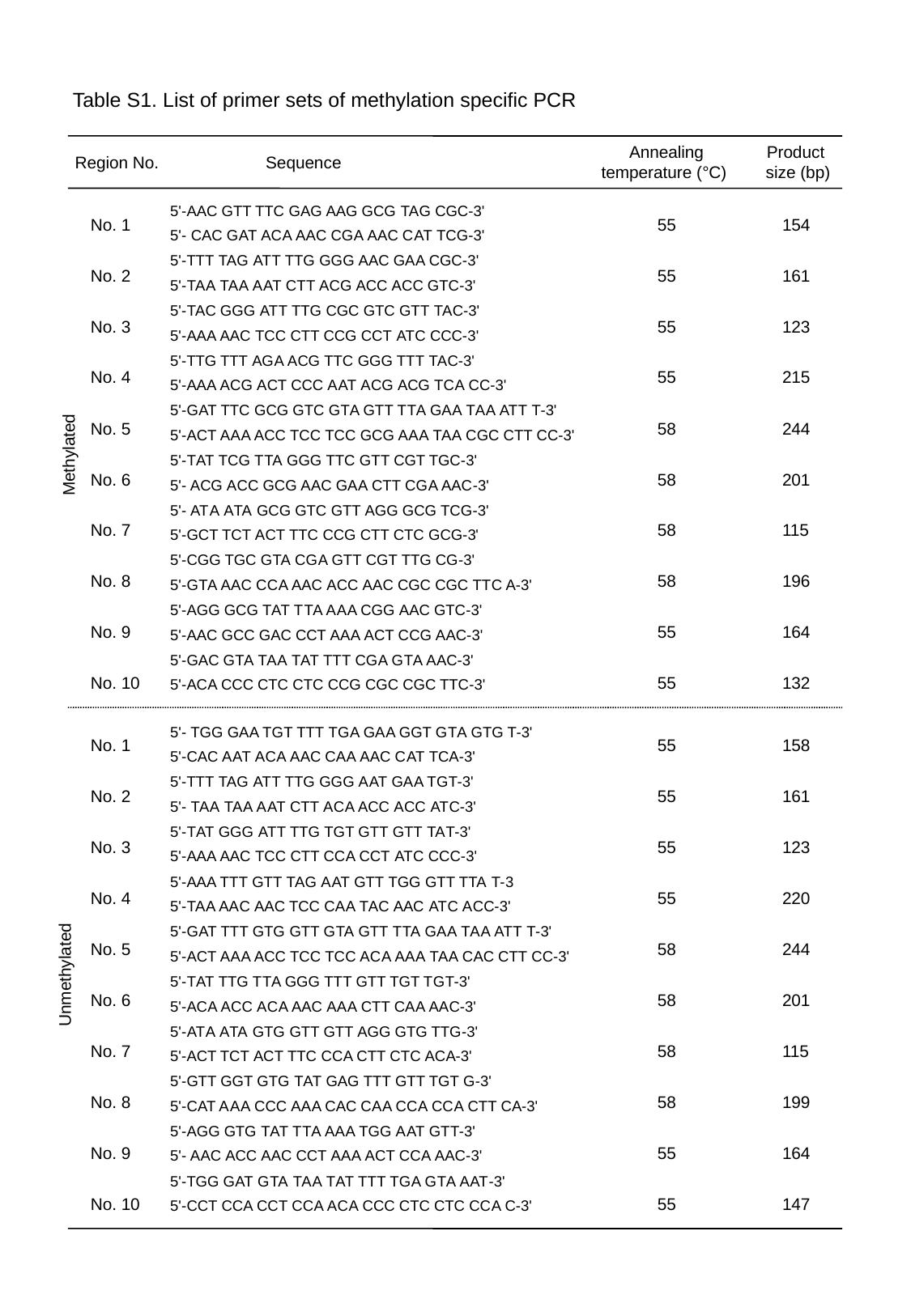

Table S1. List of primer sets of methylation specific PCR
Annealing
temperature (°C)
Product
size (bp)
Region No. Sequence
No. 1
No. 2
No. 3
No. 4
No. 5
No. 6
No. 7
No. 8
No. 9
No. 10
55
55
55
55
58
58
58
58
55
55
154
161
123
215
244
201
115
196
164
132
Methylated
No. 1
No. 2
No. 3
No. 4
No. 5
No. 6
No. 7
No. 8
No. 9
No. 10
55
55
55
55
58
58
58
58
55
55
158
161
123
220
244
201
115
199
164
147
Unmethylated
