# Supplementary material for: RUNX3 Has an Oncogenic Role in Head and Neck Cancer
Source: PLoS One. 2009 Jun 12;4(6):e5892. doi: 10.1371/journal.pone.0005892 (PMC2690822; doi:10.1371/journal.pone.0005892)
Supplement: Table S2 — (0.12 MB DOC) [file pone.0005892.s002.doc]

Table S2. List of genes up-regulated and down-regulated by Runx3 (less than 0.1) in HSC3 cells

Genes up-regulated by Runx3 (over 15-fold)

Genes down-regulated by Runx3 (less than 0.1)
