# Supplementary figures and images for: RUNX3 Has an Oncogenic Role in Head and Neck Cancer
Source: PLoS One. 2009 Jun 12;4(6):e5892. doi: 10.1371/journal.pone.0005892 (PMC2690822; doi:10.1371/journal.pone.0005892)

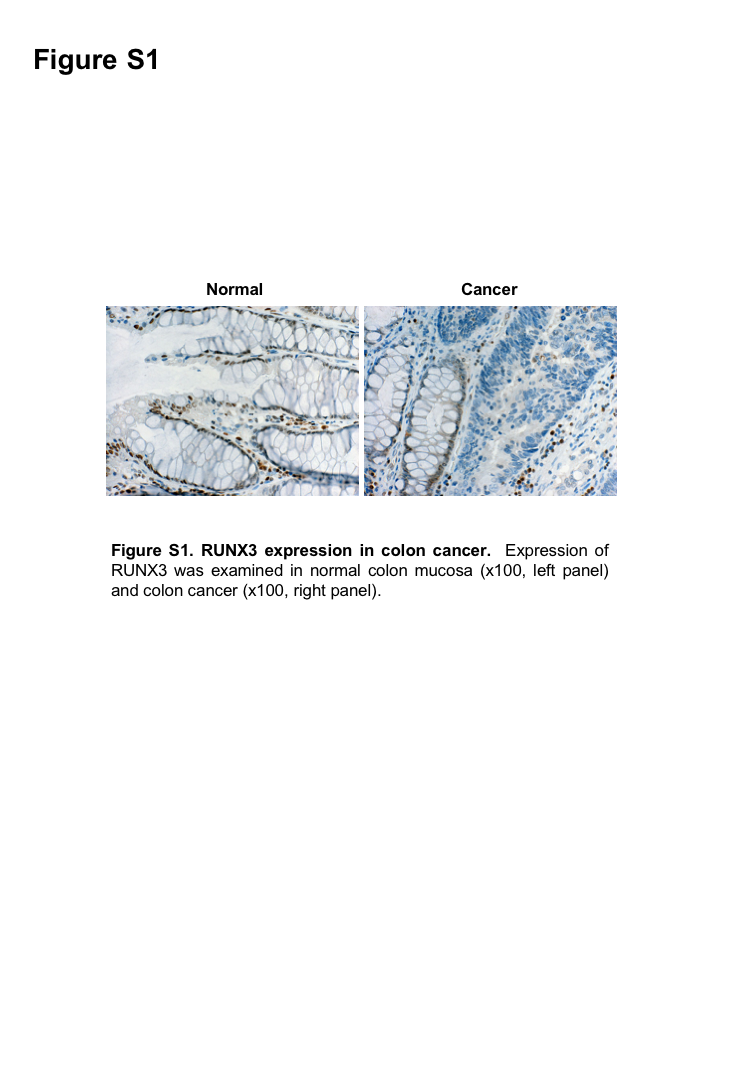

Supplement: Figure S1 — (0.35 MB TIF) [file pone.0005892.s003.tif]

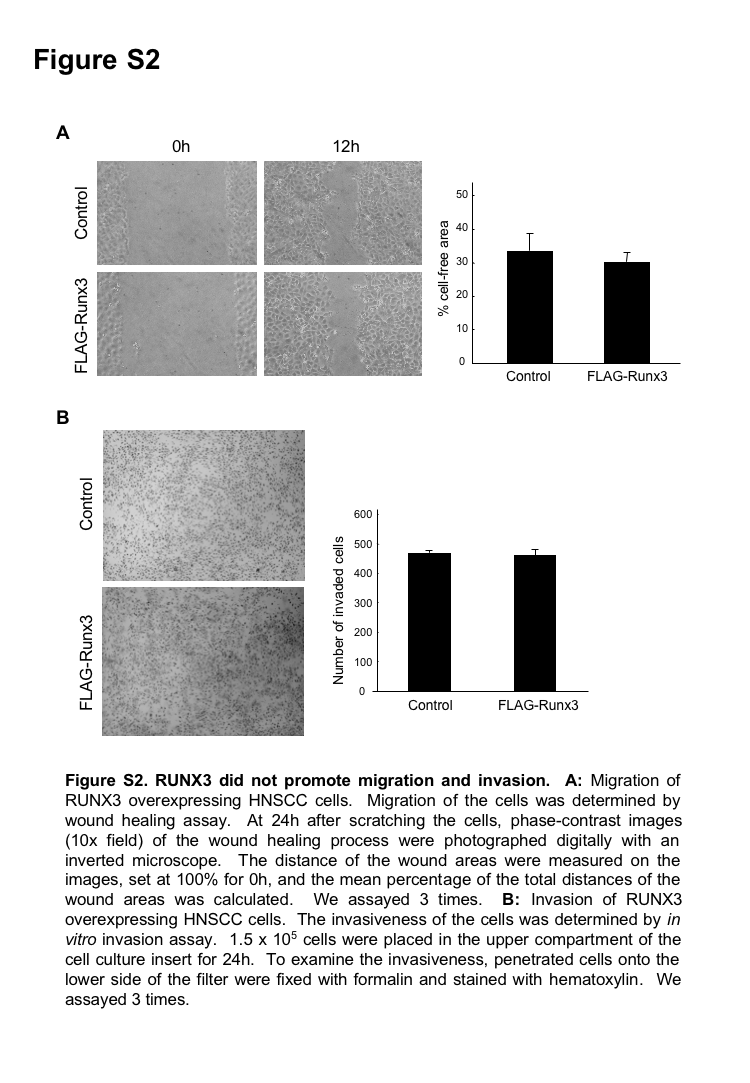

Supplement: Figure S2 — (0.39 MB TIF) [file pone.0005892.s004.tif]

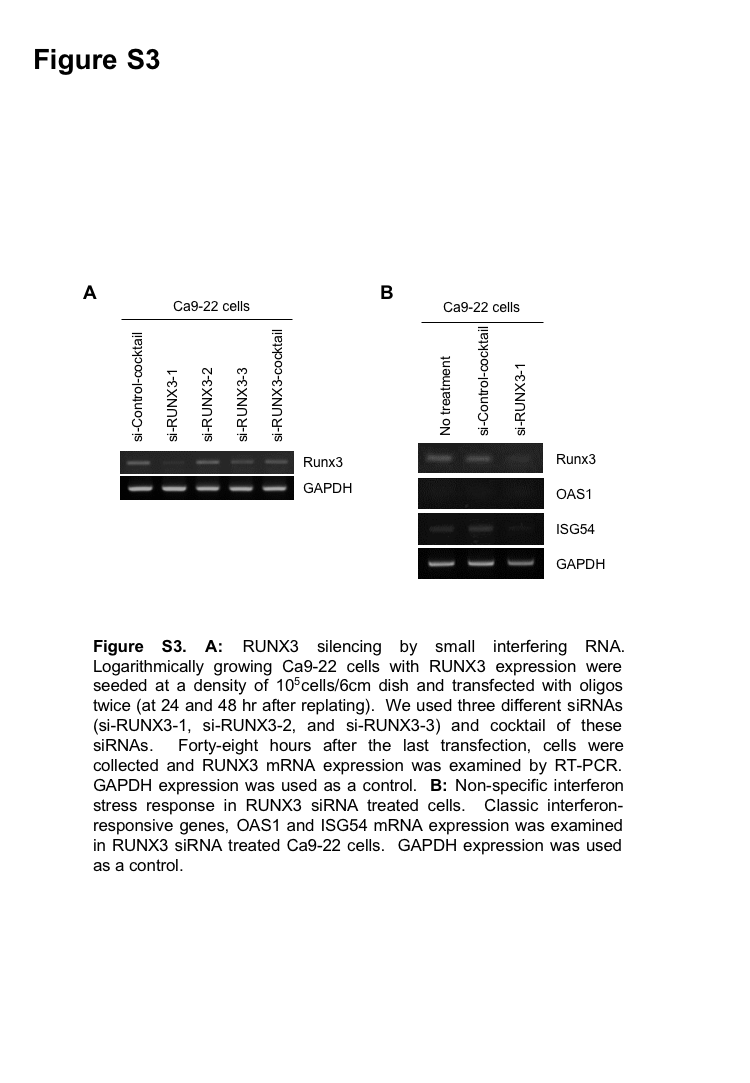

Supplement: Figure S3 — (0.17 MB TIF) [file pone.0005892.s005.tif]

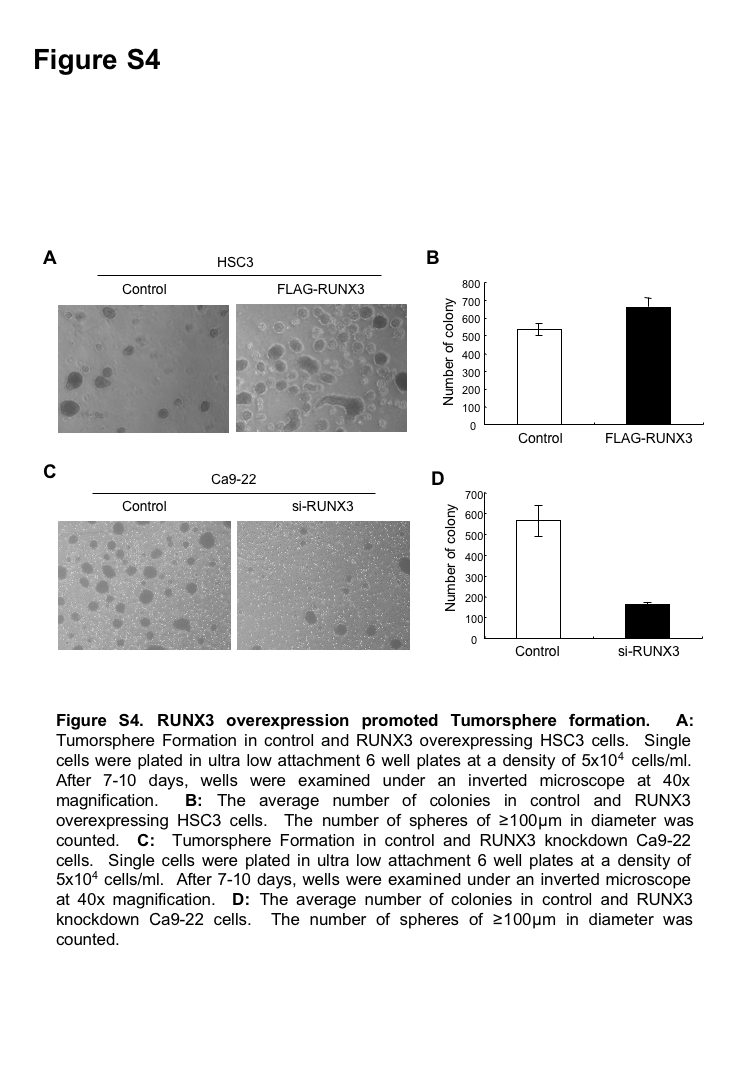

Supplement: Figure S4 — (0.31 MB TIF) [file pone.0005892.s006.tif]

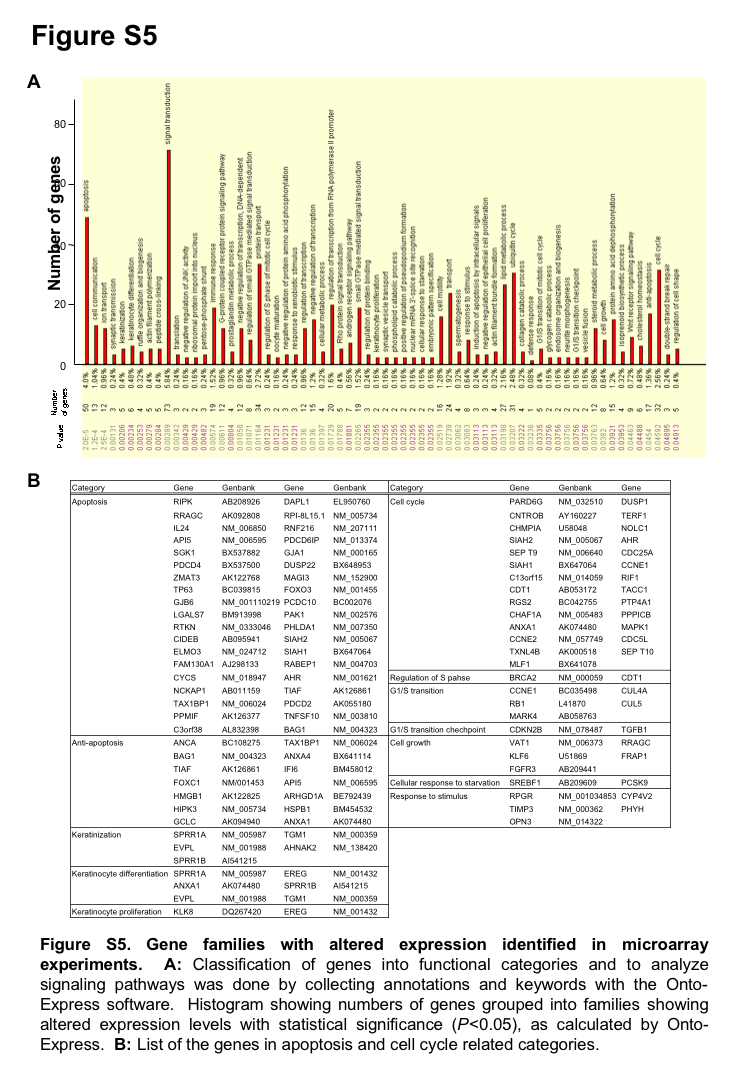

Supplement: Figure S5 — (0.90 MB TIF) [file pone.0005892.s007.tif]

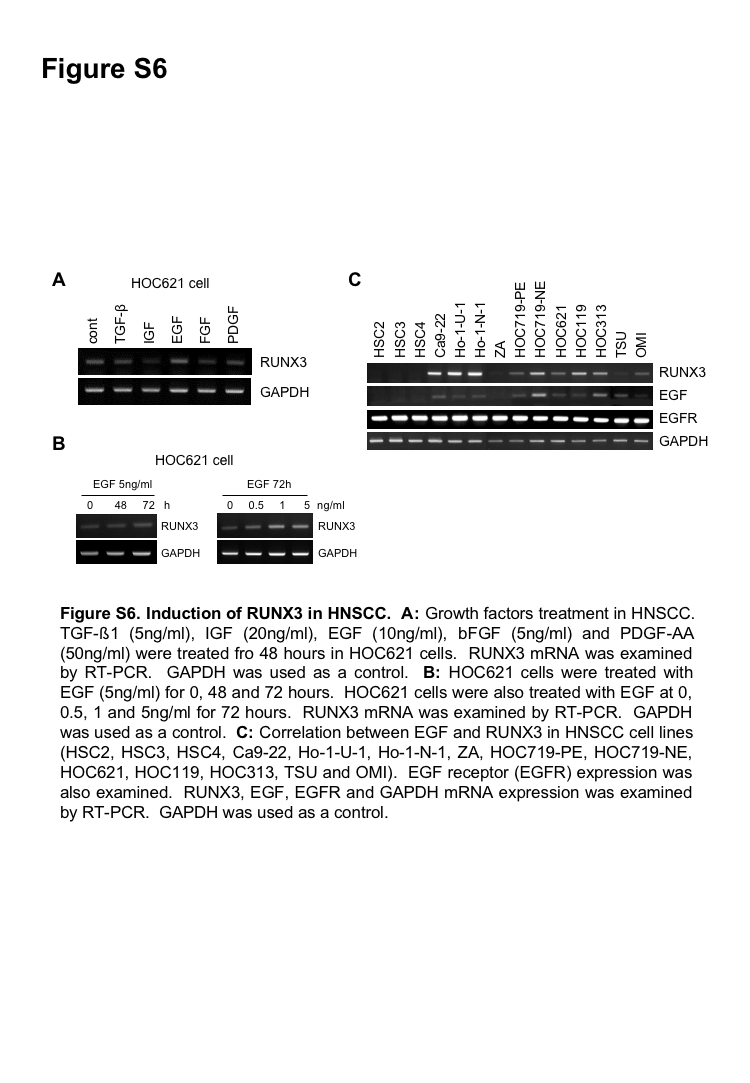

Supplement: Figure S6 — (0.20 MB TIF) [file pone.0005892.s008.tif]
